# Brain-Derived Neurotrophic Factor rs6265 polymorphism is associated with severe cancer-related fatigue and neuropathic pain in female cancer survivors

**DOI:** 10.1007/s11764-023-01426-w

**Published:** 2023-07-18

**Authors:** Taichi Goto, Diane Von Ah, Xiaobai Li, Lichen Xiang, Catherine Kwiat, Christopher Nguyen, Chao-Pin Hsiao, Leorey N. Saligan

**Affiliations:** 1 Symptoms Biology Unit, Division of Intramural Research, National Institute of Nursing Research, National Institutes of Health, Bethesda, MD USA; 2 The Ohio State University College of Nursing, Columbus, OH USA; 3 Department of Biostatistics, National Institutes of Health Clinical Center, Bethesda, MD USA; 4 Frances Payne Bolton School of Nursing, Case Western Reserve University, Cleveland, OH USA; 5 Symptoms Biology Unit, Division of Intramural Research, National Institute of Nursing Research, National Institutes of Health, 3 Center Drive, Building 3, Room 5E14, Bethesda, USA

**Keywords:** Val66Met, Fatigue, Neuropathic pain, Depression, Cancer-related symptoms, Supportive care

## Abstract

**Purpose:**

This study examined the relationships between a single-nucleotide polymorphism (SNP) of brain-derived neurotrophic factor (*BDNF*) rs6265 and psychoneurological (PN) symptoms in female cancer survivors.

**Methods:**

This secondary analysis examined 393 study participants. In addition to demographic variables, self-reported PN symptom scores (anxiety, bodily pain, depression, fatigue, neuropathic pain, and sleep disturbance) were collected using the Patient-Reported Outcomes Measurement Information System and 36-Item Short-Form Health Survey. Buccal swab samples were collected to obtain genotypes for *BDNF* rs6265 (Val/Val, Val/Met, or Met/Met). The PN symptom scores were compared across genotypes, and the relationships were examined using a regression model. We also explored correlations between different symptoms within each genotype.

**Results:**

Participants with the Met/Met genotype reported significantly worse cancer-related fatigue and neuropathic pain, which was confirmed by rank-based regression analysis. In addition, cancer-related fatigue was correlated with other PN symptoms, particularly depression. These correlations were stronger in study participants with the Met/Met genotype than those with other genotypes.

**Conclusion:**

Our study suggests that female cancer survivors with the Met/Met genotype of *BDNF* rs6265 are likely to experience worse cancer-related fatigue and neuropathic pain and that cancer-related fatigue is a good predictor of co-occurring PN symptoms in this population.

**Implications for Cancer Survivors:**

Our findings advance the scientific community's understanding of cancer-related PN symptoms experienced by female cancer survivors, especially the unique role of *BDNF* rs6265 polymorphism in these symptoms. Our findings offer valuable insights for clinical practice that the symptom experience among female cancer survivors may vary based on BDNF genotypes.

**Supplementary Information:**

The online version contains supplementary material available at 10.1007/s11764-023-01426-w.

## Introduction

Addressing the burden of cancer and cancer-treatment-related symptoms is a national research priority in the United States [1]. Even months or years after treatment completion, cancer survivors report behavioral and physical toxicities [2]. In particular, they commonly experience psychoneurological (PN) symptoms, such as cancer-related fatigue, pain, depression, anxiety, and sleep disturbance, which reduce their quality of life at disease-free stages [3]. These PN symptoms can also lead to negative health outcomes, including physical disabilities, cognitive impairments, reduced work productivity, and poor quality of life [4–8]. Studies have shown that these PN symptoms typically co-occur in cancer survivors, and cancer-related fatigue is a significant risk factor in the development of these co-occurring PN symptoms [9–12]. However, it remains unclear how these PN symptoms influence each other.

Brain-derived neurotrophic factor (BDNF) is a neurotrophin protein in the mammalian brain that plays a critical role in neuron survival and differentiation as well as synapse regulation [13, 14]. Several studies have demonstrated an association between BDNF and various PN symptoms [15–17]. Given the role of BDNF in neuronal development, differentiation, and plasticity, it is plausible that adverse symptoms, especially those associated with cancer and its treatment, may result from mutations to this neurotrophin.

The *BDNF* rs6265 polymorphism, known as Val66Met, involves the substitution of valine with methionine in the BDNF protein. This single nucleotide polymorphism (SNP) exhibits three genotypes: Val/Val (wildtype), Val/Met (heterozygote), and Met/Met (homozygote). *BDNF* rs6265 has been identified as the cause of several PN symptoms, including anxiety disorders and depression [18–21]. Interestingly, a study from 2010 showed that the Met allele increased the risk of depression in men, but not in women [22]. This disparity suggests that sex may play a role in the PN symptom experience among individuals carrying this *BDNF* rs6265 polymorphism. There are inconsistent reports regarding the associations of *BDNF* rs6265 with pain warranting further investigations. A study reported that the *BDNF* rs6265 polymorphism augmented the severity of chronic pain conditions [23]. On the other hand, Val/Val genotype showed more pain catastrophizing than the other genotypes in fibromyalgia patients [24]. *BDNF* rs6265 polymorphism have also been associated with sleep-related conditions as carriers of Met allele showed stronger forgetting of word list overnight, indicating reduced long-term memory consolidation after a night sleep [25].

Our group is investigating the relationships between the *BDNF* rs6265 polymorphism and PN symptoms among male and female cancer survivors. We have previously found that male, non-depressed cancer patients with a *BDNF* Met allele reported less cancer-related fatigue than male cancer patients with the Val/Val genotype [26]. This suggests that *BDNF* rs6265 polymorphism may have a protective advantage against cancer-related fatigue in male cancer patients. We recently observed additional evidence of *BDNF* rs6265's protective potential. When we modeled chemotherapy-induced fatigue-like behavior in mice, we found that transgenic mice homozygous for the human *BDNF* rs6265 SNP experienced fewer fatigue-like symptoms than wild-type mice [27].

However, the relationship between the *BDNF* rs6265 polymorphism and cancer-related fatigue in female cancer survivors has not been fully investigated. The present study aimed to explore this relationship in a cohort of female cancer survivors. Additionally, since cancer-related fatigue is a reported predictor of many co-occurring PN symptoms [11], understanding the relationship of *BDNF* rs6265 polymorphism with cancer-related fatigue will elucidate the potential genomic mechanisms underpinning the co-occurrence of cancer-related PN symptoms.

## Methods

### Study participants

This study is a secondary analysis of the data obtained from a larger investigation examining factors associated with cancer-related cognitive impairment in male and female breast and colorectal cancer survivors (ClinicalTrials.gov Identifier: NCT04611620). Breast and colorectal cancer survivors were recruited through Institutional Review Board-approved advertisements via social media (i.e., Facebook) and online cancer-affiliated resource sites (e.g., Pink Ribbon Connection, Dr. Susan Love Foundation, and Colorectal Cancer Alliance, etc.).

For the parent study, cancer survivors aged 21 years or older were enrolled if they were ≥ 6 months post-adjuvant therapy and neo-adjuvant therapy for early-stage (Stage I–III) breast or colorectal cancer (except for breast cancer survivors on Aromatase Inhibitors or Tamoxifen), reported cancer-related cognitive concerns (e.g., memory issues, attention problems), and ability to provide written consent and HIPAA authorization. Participants were excluded if they reported metastatic breast or colorectal cancer (Stage IV) at time of consenting, or if they were unable to read and understand English to complete survey questionnaires. In this sub-study, only female cancer survivors were included in the analysis.

### Ethical considerations

Participants completed written online informed consent through the HIPAA-approved REDCap® form prior to study entry. The study was approved in 2020 by a large comprehensive cancer center in the Midwest and the Indiana University Institutional Review Board (Protocol Number: NURS-IIR-IUSCCC-0748).

### Data collection

After obtaining patient consent, participants were given a unique patient identifier and completed an individualized series of demographic forms and Patient-Reported Outcomes Measurement Information System (PROMIS) questionnaires (see list below) via REDCap®. Additionally, cognitive performance was assessed using the Cambridge Neuropsychological Test Automated Battery (CANTAB^©^) (Cambridge Cognition Ltd., Cambridge, United Kingdom).

Buccal swab samples were collected using the OmniSwab kit (Catalog Number WB100035, Qiagen, Germantown, Maryland, US). Each OmniSwab kit and was mailed to study participants for self-collection. Participants returned samples via mail no more than 2 days after sample collection. The research team provided reminder calls to participants to answer any questions pertaining to sample collection.

Demographic information, medical history, and treatment characteristics were collected through an investigator-initiated sociodemographic form. Using this form, participants reported their demographic characteristics, including age, race/ethnicity, marital status, educational history, employment status, income, type of cancer (breast or colorectal cancer), diagnosis date, cancer stage at diagnosis, and cancer treatment (chemotherapy, surgery, and/or radiation therapy).

PN symptoms were assessed using PROMIS short forms, which included cancer-related fatigue [28], peripheral neuropathy, depression [29], anxiety [29], and sleep disturbance [30]. Each 8-item PROMIS short form was rated on a 5-point Likert scale from 0 to 4, with higher scores indicating worse symptomology (greater cancer-related fatigue, neuropathic pain, depression, anxiety, and sleep disturbance). The pain was assessed using the 2-item bodily pain subscale of the 36-Item Short-Form Health Survey (SF-36), a structured, self-report questionnaire [31]. The SF-36 has been shown to be a comprehensive measure of general health that has shown reliability and validity in various populations including cancer patients.

### Brain-Derived Neurotrophic Factor rs6265 polymorphism genotyping

Upon receipt, participant buccal swab samples were stored at -80 °C and were batch-shipped in ice to Genewiz (Azenta Life Sciences, South Plainfield, New Jersey, US) for processing. Briefly, DNA was extracted using the QIAamp® DNA Mini Kit (QIAGEN, Germantown, Maryland, US) following protocols included in the kit. The region containing the *BDNF* rs6265 (GenBank dbSNP: rs6265) was profiled using polymerase chain reaction (PCR) with the following primers: forward 5’–AGAAGAGGAGGCTCCAAAGG–3’ and reverse 5’–ACAAGGTGGCTTGGCCTAC–3’. After enzymatic purification, sequencing was performed using the BigDye™ Terminator Cycle Sequencing Kit (ThermoFisher Scientific, Waltham, Massachusetts, US). Data analysis was performed using the DNASTAR Lasergene12® software (DNASTAR, Inc, Madison, WI, US). The threshold for SNP detection was set at 10%. Mutations from the reference sequence were called when the sequence quality and coverage were sufficient.

### Statistical analyses

Descriptive statistics were reported for each variable. Normally distributed variables were presented as means and standard deviations (SD), other continuous variables were presented as medians and interquartile ranges (IQR), and categorical variables were presented as numbers and percentages.

Study participants were clustered based on their PN symptom scores and a heatmap profile was generated using hierarchical clustering. The pheatmap R package (version 1.0.12) was used to divide the participants into two cluster groups. *BDNF* genotype frequency in each cluster group  were then compared using the Fisher's exact test [32].

The Kruskal–Wallis (KW) test was used to compare each PN symptom score across the *BDNF* genotype groups. Then, *post hoc* Wilcoxon rank-sum tests were conducted to assess the pairwise comparisons. A sample size calculation and power analysis were not performed for this sub-analysis, since this is a hypothesis-generating study conducting secondary analysis of the data already collected from the parent study. To avoid the loss of clinically meaningful results, we reported raw *p*-values without adjustments for the *post hoc* multiple pairwise comparisons.

For the PN symptom scores that showed a genotype difference, multivariable regression analyses were conducted to explore the effects of potential confounders (demographic and clinical variables) on the outcome variables in addition to the *BDNF* rs6265 genotypes. Co-explanatory variables and final models for each outcome were determined based on univariable regression models, the drop in dispersion test, and the R^2^ values of each model. Since the assumptions of the linear regression analysis did not hold for the selected PN symptom scores, rank-based regression was used to analyze the data using the Rfit R package (version 0.24.2) [33]. Spearman’s correlation coefficients were calculated to examine the relationships between each pair of the PN symptom scores. A *p*-value less than 0.05 was considered statistically significant. The statistical analyses were performed using R statistical software (version 4.2.1).

## Results

### Sample description

A total of 394 female cancer survivors were included in this analysis, but one study participant was excluded due to a lack of *BDNF* genotypic data. Therefore, the final analysis included 393 study participants. Demographic data, as shown in Table 1, indicated that the mean age of the study participants was 55.1 years ± 9.8 (mean ± SD). The median (IQR) years since cancer diagnosis was 4 (6). Additionally, 365 (93%) were White, 356 (91%) had breast cancer, and 37 (9%) had colon or rectal cancer. Of the 393 study participants, 258 (65.6%) had Val/Val, 123 (31.3%) had Val/Met, and 12 (3.1%) had Met/Met genotypes; in terms of allele frequency, 639 (81.3%) had the Val allele, and 154 (18.7%) had the Met allele (Supplementary Table S1). These genotype distributions are consistent with the expected genotype distribution for an overwhelmingly homogenous sample (93% White) based on a previous study [34].

**Table 1:** Demographic data of female cancer survivors analyzed in this study (*n* = 393)

		Val/Val (*n* = 258)	Val/Met (*n* = 123)	Met/Met (*n* = 12)	*P*-value	Test
Age (years), mean (SD)		55.3 (9.60)	54.9 (10.4)	54.4 (9.60)	.92	a
Years since cancer diagnosis (years), median (IQR)		4 (6)	3 (5)	3.5 (7.5)	.26	k
Race	White	236 (91)	120 (98)	9 (75)	**.005**	f
	Others	22 (9)	3 (2)	3 (25)		
Marital status	Living with someone	195 (76)	92 (75)	6 (50)	.16	f
	Others	63 (24)	31 (25)	6 (50)		
Education history	Highschool/Undergraduate/Associate	164 (64)	79 (64)	9 (75)	.77	f
	Master's/PhD	94 (36)	44 (36)	3 (25)		
Employment status*	Working	170 (68)	83 (68)	5 (50)	.71	f
	Retired	60 (24)	31 (26)	4 (40)		
	Unemployed	20 (8)	7 (6)	1 (10)		
Income*	< $75 K	95 (41)	38 (37)	6 (60)	.07	c
	> $75 K	136 (59)	64 (63)	4 (40)		
Cancer type	Breast Cancer	232 (90)	112 (91)	12 (100)	.75	f
	Colon/Rectal Cancer	26 (10)	11 (9)	0 (0)		
Cancer stage	I	78 (30)	43 (35)	1 (8)	**.02**	f
	II	117 (45)	45 (37)	3 (25)		
	III	63 (24)	35 (28)	8 (67)		
Chemotherapy	Yes	230 (89)	111 (90)	12 (100)	.76	f
Surgery	Yes	256(99)	123 (100)	12 (100)	1	f
Radiation	Yes	180 (70)	82 (67)	12 (100)	**.04**	f

"Others" in race includes "American Indian or Alaskan Native," "Asian," "Black," and "More than one race." "Living with someone" in marital status includes "Living with partner," and "married." "Highschool/Undergraduate/Associate" in educational history includes "Highschool graduate," "Undergraduate/Bachelor's degree or equivalent" and "Associate's degree/some college;" Master's/PhD includes "Master's degree or equivalent" and "PhD or equivalent." "Working" in employment status includes "Full-time (> 35 h/wk)," "Homemaker," and "Part-time (< 20 h/wk)." * The study participants who answered "other" or did not answer to the questions were excluded. a, ANOVA; k, Kruskal–Wallis test; f, Fisher's exact test; c, chi-squared test. *P*-values shown in bold indicate statistical significance

### Study participant clustering and *BDNF* genotype frequency

**Fig. 1:**
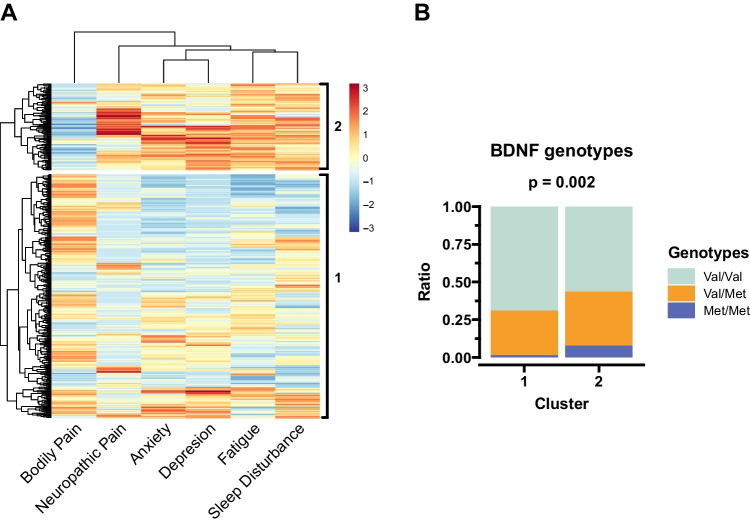
Clustering the subjects based on their PN symptom scores and the *BDNF* predispositions in each cluster. **A**) The study participants were clustered using hierarchical clustering based on their PN symptom scores and divided into two groups exploratorily. **B**) The *BDNF* genotype frequencies were compared between the two clusters using Fisher’s exact test

To identify patient clusters based on cancer related PN symptoms, we employed a hierarchical clustering method based on symptom scores. Figure 1A shows the clustered heatmap profiles of the symptom scores reported by study participants. This method identified two symptom cluster groups, where cluster group 2 had higher (more severe) PN symptom scores except for bodily pain. Additionally, *BDNF* genotype frequencies were different between the symptom cluster groups; where cluster group 2 had more Met/Met genotype participants than cluster group 1 (Fig. 1B).

### Relationships between the *BDNF* genotypes and PN symptoms

**Fig. 2:**
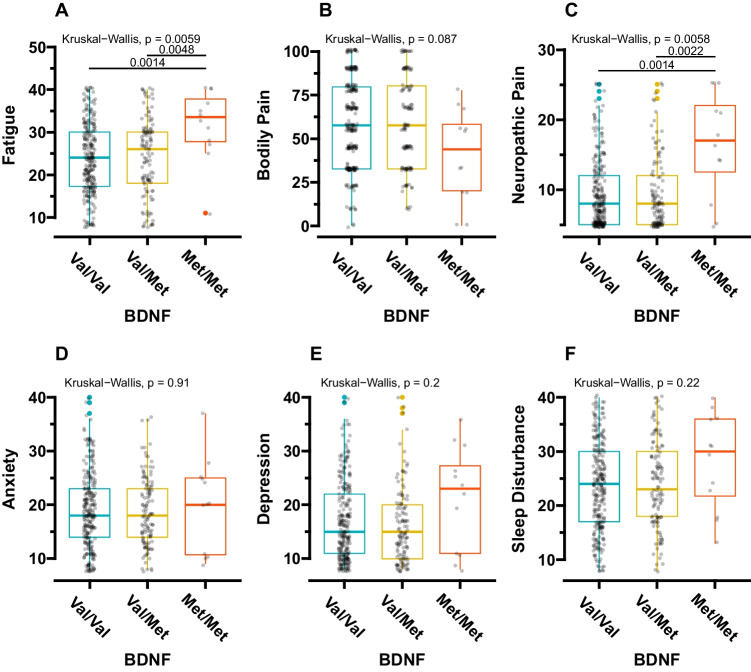
The differences in each PN symptom per *BDNF* genotype. The Kruskal-Wallis test and *post-hoc* multiple comparisons using Wilcoxon’s rank-sum test were applied to all the statistical comparisons. BDNF, brain-derived neurotrophic factor

Figure 2 displays all inter-genotype comparisons for each PN symptom, revealing significant differences in cancer-related fatigue (Kruskal–Wallis, *p* = 0.006) (Fig. 2A) and neuropathic pain scores (Kruskal–Wallis, *p* = 0.006) (Fig. 2C). Post-hoc pairwise comparison tests indicated that participants with the Met/Met genotype reported more severe cancer-related fatigue than those with the Val/Val (*p* = 0.001) and Val/Met (*p* = 0.005) genotypes, respectively (Fig. 2A). Additionally, study participants with the Met/Met genotype reported significantly higher neuropathic pain than those with the Val/Val (*p* = 0.001) and Val/Met (*p* = 0.002) genotypes (Fig. 2C).

Since the frequencies of White participants, clinical cancer stages, and prior treatment with radiation therapy were different among the genotypes, we conducted sub-group analyses. The study participants with the Met/Met genotype had the most severe cancer-related fatigue and neuropathic pain scores (1) among White participants, (2) in participants with clinical stage III cancer, and (3) among those who underwent radiation therapy (Supplementary Fig. S1–3). Among White participants, those with the Met/Met genotype also had the most severe bodily pain compared to participants with other genotypes (Supplementary Fig. S1).

### Exploratory regression analysis using rank regression model for cancer-related fatigue and neuropathic pain

As mentioned above, cancer-related fatigue and neuropathic pain scores were selected as response variables for multivariable regression analyses using the rank-based regression model. The cancer-related fatigue score was associated with the *BDNF* rs6265 Met/Met genotype (slope = 6.00, *p* = 0.048), years since cancer diagnosis (slope = -0.19, *p* = 0.03), those with a master’s/PhD (slope = -2.73, *p* = 0.01), and those who are unemployed (slope = 4.84, *p* = 0.01). This analysis revealed that those with the Met/Met genotype had 6.00 points higher (worse) cancer-related fatigue scores than those with the Val/Val genotype. Neuropathic pain score was associated with the *BDNF* rs6265 Met/Met genotype (slope = 9.30, *p* < 0.001), years since cancer diagnosis (slope = -0.08, *p* = 0.02), and having a master’s/PhD (slope = -1.00, *p* = 0.01). The final models are shown in Supplementary Table S2.

### Cancer-related fatigue as a predictor for other PN symptoms

**Fig. 3:**
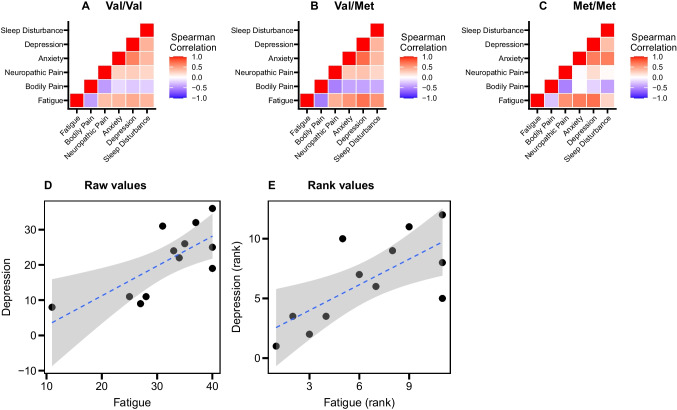
Correlations between each PN symptom. Spearman’s correlation was applied. **A**–**C**) Correlation matrices of all study participants in each genotype group. **D**) A scatter plot between absolute values of the fatigue score and depression score in the study participants with Met/Met genotype. **E**) A scatter plot between ranked values of the fatigue score and depression score in the study participants with Met/Met genotype

We calculated correlation coefficients between symptom scores for each genotype to examine the role of cancer-related fatigue as a predictor of other PN symptoms. In participants with the Val/Val genotype, cancer-related fatigue scores were moderately correlated with bodily pain (*ρ* = -0.42) and depression (*ρ* = 0.45), (Fig. 3A and Supplementary Table S3). In participants with the Val/Met genotype, cancer-related fatigue scores were correlated with all other PN symptoms, and highly correlated with depression (*ρ* = 0.70) (Fig. 3B and Supplementary Table S3). In participants with the Met/Met genotype, although the correlation coefficients of cancer-related fatigue with bodily pain and sleep disturbance were lower compared to those with other genotypes, the correlation of cancer-related fatigue with neuropathic pain (*ρ* = 0.57), anxiety score (*ρ* = 0.67), and depression (*ρ* = 0.71) were higher than those with other genotypes (Fig. 3C and Supplementary Table S3).

## Discussion

In the present study, we investigated the association between the *BDNF* rs6265 polymorphism and PN symptoms in 393 female breast and colorectal cancer survivors. Our study showed that participants with the Met/Met genotype had more severe cancer-related fatigue and neuropathic pain than those with the other genotypes. These findings were consistent even after the sub-group analyses of White participants, participants with clinical cancer stage III, and those who received prior radiation therapy although these three variables were significantly different between the genotypes. The relationships of *BDNF* rs6265 polymorphism with cancer-related fatigue and neuropathic pain were also confirmed by multivariable rank-based regression. Finally, we showed that cancer-related fatigue may be a predictor of other co-occurring PN symptoms. Specifically, correlations of cancer-related fatigue with neuropathic pain, anxiety, and depression were higher in participants with the Met/Met genotype than in those with other genotypes. These results suggest that *BDNF* polymorphism-based, patient-focused therapeutic strategies for symptom management may effectively improve health outcomes for cancer survivors.

Our previous study found that male, non-depressed cancer patients with the Met carrier for *BDNF* rs6265 polymorphism experienced a protective advantage against cancer-related fatigue [26]. We also conducted an animal study and observed that female mice were less fatigued than male mice, regardless of genotype [27]. However, in the current study, we observed that female breast and colorectal cancer survivors with the Met/Met genotype reported more severe cancer-related fatigue compared to those with other genotypes. This suggests a sex variation in the protective advantage conferred by the *BDNF* rs6265 polymorphism. Previous studies have documented sex variations in symptom reporting, especially for cancer-related fatigue with females reporting higher cancer-related fatigue severity than males [35]. Sex variation has also been reported for BDNF serum levels in depressed patients, with females having lower levels than males [36]. Additionally, an increase in serum BDNF concentration in Met allele carriers of the *BDNF* rs6265 polymorphism was found to be specific to the male population [37].

Previously, we reported that lower BDNF serum levels were associated with worsening cancer-related fatigue during radiation therapy in prostate cancer patients [17]. Our current findings expand on these results, proposing a new hypothesis that the influence of the *BDNF* polymorphism on cancer-related fatigue severity depends on sex. In female cancer survivors carrying the *BDNF* Met/Met genotype, lower BDNF serum levels result in more severe fatigue. Our current finding provides early evidence of potential sex variations in the influence of *BDNF* polymorphism on the cancer-related fatigue experience. However, considering the small number of participants with the Met/Met genotype in our cohort, further investigation is required. If confirmed, the knowledge of sex variation in the fatigue-protective advantage of the *BDNF* polymorphism would help advance therapeutic strategies for symptom management to improve health outcomes for cancer survivors.

Another significant finding of the study is that participants with the Met/Met genotype reported more severe neuropathic pain than those with other genotypes. BDNF plays a crucial role in neuroprotection, neuronal differentiation, and neuronal regeneration through various pathways [38]. The *BDNF* rs6265 polymorphism was first reported in 2003 by Egan and colleagues [39], who associated it with various psychiatric and neurological disorders and diseases [20, 40]. Increasing evidence suggests that this *BDNF* polymorphism contributes to differences in pain perception. For instance, an earlier study reported that the *BDNF* rs6265 polymorphism augmented the severity of chronic pain conditions [23]. Conversely, another study showed that individuals with the Met allele had a lower risk of post-surgical pain [41]. This dual effect of the *BDNF* rs6265 polymorphism aligns with our findings, showing that individuals with the Met/Met genotype had more severe neuropathic pain but less severe bodily pain than those with the other two genotypes.

The current study confirms the relationship between cancer-related fatigue and other co-occurring PN symptoms. Especially, participants with the Met allele (those with the Val/Met and Met/Met genotypes) had higher correlation coefficients between cancer-related fatigue and other PN symptoms. Among participants with the Met/Met genotype, cancer-related fatigue was highly correlated with depression. A previous study reported that cancer-related fatigue often predicted subsequent depression, insomnia, and pain in cancer patients followed longitudinally [11]. While some studies have linked the *BDNF* rs6265 polymorphism with depression [42–44], our study did not find a difference in the depression scores between the three *BDNF* genotypes. However, a 2013 meta-analysis found an association between *BDNF* rs6265 and depression [45], and a 2010 meta-analysis reported an increased risk of depression among male Met carriers [22]. Our results suggest that cancer-related fatigue predicts depression, especially in participants with the *BDNF* Met/Met genotype, and the relationship between the *BDNF* polymorphism and depression appears to be mediated by cancer-related fatigue in our population of female cancer survivors. Because of our cross-sectional study design, we were unable to determine causal relationships between *BDNF* polymorphism and PN symptoms. Future longitudinal studies should be conducted to explore these relationships.

Our current study had several limitations. First, the small sample size of only 12 study participants with the Met/Met genotype may have led to a type II statistical error. Thus, some results were not statistically significant, although we observed trends toward a statistical difference such as bodily pain scores. However, the genotype and allele frequency in this study were comparable to our previous study involving male cancer patients and to globally conducted research enrolling multiple races and populations [26, 46]. Second, the racial composition of our study participants was overwhelmingly White (93%). The frequency of the Met allele of *BDNF* rs6265 polymorphism varies among populations, where those of Asian descent tend to carry the Met allele more (up to 72%) than other races [46]. In addition, racial differences in symptom reporting are well documented [47]. Therefore, further studies should enroll sufficient numbers of study participants from multiple racial categories to increase the generalizability of our findings.

In conclusion, this study suggested potential sex variations in the protective effects of the *BDNF* rs6265 polymorphism against cancer-related fatigue. Specifically, female breast and colorectal cancer survivors carrying the Met/Met genotype experienced more severe cancer-related fatigue and neuropathic pain. These findings offer valuable insights for clinical practice, suggesting that the experience of symptoms among cancer survivors may vary based on sex and can be influenced by *BDNF* genotypes. This underscores the need for tailored approaches in the assessment and management of symptoms in our clinical practice. The findings also suggest a need for further research into the biological and psychological mechanisms underlying these symptoms. Our study will stimulate future research investigating the underlying mechanisms of these distressing cancer-related psychoneurological symptoms and potential interventions to manage them.

## Supplementary Information

Below is the link to the electronic supplementary material.

Supplementary file1 (DOCX 326 KB)

## Data Availability

The datasets generated during and/or analyzed during the current study are available from the corresponding author upon reasonable request.
