# Correction to: Brain-Derived Neurotrophic Factor rs6265 polymorphism is associated with severe cancer-related fatigue and neuropathic pain in female cancer survivors

**DOI:** 10.1007/s11764-026-02015-3

**Published:** 2026-04-15

**Authors:** Taichi Goto, Diane Von Ah, Xiaobai Li, Lichen Xiang, Catherine Kwiat, Christopher Nguyen, Chao-Pin Hsiao, Leorey N. Saligan

**Affiliations:** 1 Symptoms Biology Unit, Division of Intramural Research, National Institute of Nursing Research, National Institutes of Health, Bethesda, MD USA; 2 Center for Healthy Aging, Self-Management & Complex Care, College of Nursing, The Ohio State University, Columbus, OH USA; 3 Department of Biostatistics, National Institutes of Health Clinical Center, Bethesda, MD USA; 4 Case Western Reserve University School of Nursing, Cleveland, OH USA

After publication of the original article [1], we identified an error in the description and interpretation of the direction of the bodily pain scores derived from the 2-item Bodily Pain subscale of the 36-Item Short-Form Health Survey (SF-36) [2]. According to the SF-36 scoring instructions, responses are transformed to a 0–100 scale, with higher scores indicating less bodily pain (better health status) and lower scores indicating more bodily pain (worse health status). In the originally published article, we inadvertently interpreted higher Bodily Pain scores as indicating more severe pain, which is the opposite of the SF-36 scoring direction.

This error does not affect the results of the statistical analyses, including the *p*-values, because the analyses were conducted using the correctly scored SF-36 Bodily Pain values. We have corrected the relevant text in the “Methods,” “Results,” and “Discussion” sections to reflect the proper interpretation of the SF-36 Bodily Pain scale.

## Methods

### Data collection

The pain was assessed using the 2-item bodily pain subscale of the 36-Item Short-Form Health Survey (SF-36), a structured, self-report questionnaire [2]. Responses are transformed to a 0–100 scale, with higher scores indicating less bodily pain (better health status) and lower scores indicating more bodily pain (worse health status). The SF-36 has been shown to be a comprehensive measure of general health that has shown reliability and validity in various populations including cancer patients.

## Results

### Study participant clustering and BDNF genotype frequency

Figure 1A shows the clustered heatmap profiles of the symptom scores reported by study participants. This method identified two symptom cluster groups, where cluster group 2 had more severe PN symptoms.

## Discussion

Conversely, Tian et al. reported that carriers of the Met allele had a lower risk of chronic postsurgical pain [3]. At face value, this appears inconsistent with our findings, in which individuals with the Met/Met genotype had more severe neuropathic pain and lower SF-36 Bodily Pain scores (i.e., greater bodily pain) than those with the other two genotypes. However, Tian et al. also suggested that the risk of chronic postsurgical pain may vary by sex, and population differences in rs6265 associations have been reported. For example, the rs6265 Met/Met genotype was associated with lower cognitive abilities in a cohort with a high proportion of White breast cancer survivors, whereas it was reported as protective in Asian breast cancer survivors [4, 5]. Such heterogeneity in the role of rs6265 across sex and populations may contribute to variation in pain symptom phenotypes and warrants further investigation.
