# Supplementary material for: Brain-Derived Neurotrophic Factor rs6265 polymorphism is associated with severe cancer-related fatigue and neuropathic pain in female cancer survivors
Source: J Cancer Surviv. 2023 Jul 18;18(6):1851–60. doi: 10.1007/s11764-023-01426-w (PMC11502548; doi:10.1007/s11764-023-01426-w)
Supplement: Supplementary file 1 — Supplementary file1 (DOCX 326 KB) [file 11764_2023_1426_MOESM1_ESM.docx]

**Supplementary materials**

**Title:**

Brain-Derived Neurotrophic Factor rs6265 polymorphism is associated with severe cancer-related fatigue and neuropathic pain in female cancer survivors.

**Journal:**

Journal of Cancer Survivorship

**Authors:**

Taichi Goto^1^, Diane Von Ah^2^, Xiaobai Li^3^, Lichen Xiang^1^, Catherine Kwiat^1^, Christopher Nguyen^1^, Chao-Pin Hsiao^4^, Leorey N. Saligan^1*^

1. Symptoms Biology Unit, Division of Intramural Research, National Institute of Nursing Research, National Institutes of Health, Bethesda, MD, USA
2. The Ohio State University College of Nursing, Columbus, OH, USA
3. Department of Biostatistics, National Institutes of Health Clinical Center, Bethesda, MD, USA
4. Case Western Reserve University School of Nursing, Cleveland, OH, USA

*** Corresponding Author:**

Leorey N. Saligan, PhD

3 Center Drive, Building 3, Room 5E14, Bethesda, MD 20892, USA

301-451-1685

[saliganl@mail.nih.gov](mailto:saliganl@mail.nih.gov)

**Supplementary materials**

**Figure S1.** Differences in each PN symptom per BDNF genotype among White study participants.

The Kruskal-Wallis test and post-hoc multiple comparisons using Wilcoxon’s rank-sum test were applied to all statistical comparisons.

BDNF, brain-derived neurotrophic factor

**Figure S2.** Differences in each PN symptom per BDNF genotype in the study participants with clinical cancer stage III of both breast and colon/rectal cancer types.

The Kruskal-Wallis test and post-hoc multiple comparisons using Wilcoxon’s rank-sum test were applied to all statistical comparisons.

BDNF, brain-derived neurotrophic factor

**Figure S3.** Differences in each PN symptom per BDNF genotype in post-radiation therapy study participants.

The Kruskal-Wallis test and post-hoc multiple comparisons using Wilcoxon’s rank-sum test were applied to all statistical comparisons.

BDNF, brain-derived neurotrophic factor

| **Table S1.** Genotype and allele frequencies of the BDNF Val66Met polymorphism | | |
| --- | --- | --- |
| Genotype | Absolute frequency (#) | Relative frequency % |
| C/C (Val/Val) | 258 | 65.6 |
| C/T (Val/Met) | 123 | 31.3 |
| T/T(Met/Met) | 12 | 3.1 |
| C (Val) allele | 639 | 81.3 |
| T (Met) allele | 147 | 18.7 |
|  | | |

| **Table S2.** Multivariate rank-based regression models for all study participants | | | | |
| --- | --- | --- | --- | --- |
|  |  | Coefficients | SE | *p-*value |
| Fatigue | |  |  |  |
|  | BDNF Val/Met | 1.11 | 1.01 | .22 |
|  | BDNF Met/Met | 6.00 | 3.03 | **.048** |
|  | Years Since Cancer Diagnosis | -0.19 | 0.08 | **.03** |
|  | Education History - Master's/PhD | -2.73 | 0.98 | **.01** |
|  | Employment - Retired | -1.67 | 1.12 | .14 |
|  | Employment - Unemployment | 4.84 | 1.81 | **.01** |
| Neuropathic Pain | |  |  |  |
|  | BDNF Val/Met | -0.17 | 0.42 | .70 |
|  | BDNF Met/Met | 9.30 | 1.27 | **< .001** |
|  | Years Since Cancer Diagnosis | -0.08 | 0.03 | **.02** |
|  | Education History - Master's/PhD | -1.00 | 0.41 | **.01** |
| Rank-based regression was employed. The reference groups for BDNF, education history, and employment were “Val/Val,” “Highschool/Undergraduate/Associate,” and “Working,” respectively. | | | | |

| **Table S3** Correlation matrix | | | | | | |
| --- | --- | --- | --- | --- | --- | --- |
|  |  | Bodily Pain | Neuropathic Pain | Anxiety Score | Depression | Sleep Disturbance |
| Val/Val | Fatigue | -0.42 | 0.34 | 0.38 | 0.45 | 0.38 |
|  | Bodily Pain |  | -0.43 | -0.17 | -0.24 | -0.21 |
|  | Neuropathic Pain |  |  | 0.24 | 0.26 | 0.22 |
|  | Anxiety Score |  |  |  | 0.64 | 0.37 |
|  | Depression |  |  |  |  | 0.40 |
|  |  |  |  |  |  |  |
| Val/Met | Fatigue | -0.56 | 0.41 | 0.55 | 0.70 | 0.55 |
|  | Bodily Pain |  | -0.55 | -0.39 | -0.40 | -0.37 |
|  | Neuropathic Pain |  |  | 0.29 | 0.32 | 0.22 |
|  | Anxiety Score |  |  |  | 0.74 | 0.36 |
|  | Depression |  |  |  |  | 0.39 |
|  |  |  |  |  |  |  |
| Met/Met | Fatigue | -0.26 | 0.57 | 0.67 | 0.71 | 0.22 |
|  | Bodily Pain |  | -0.54 | 0.01 | -0.19 | -0.41 |
|  | Neuropathic Pain |  |  | -0.02 | 0.18 | 0.03 |
|  | Anxiety Score |  |  |  | 0.65 | 0.65 |
|  | Depression |  |  |  |  | 0.34 |
| Spearman's correlation was applied. | | | | | |  |
